# Rainfall Prediction System Using Machine Learning Fusion for Smart Cities

**DOI:** 10.3390/s22093504

**Published:** 2022-05-04

**Authors:** Atta-ur Rahman, Sagheer Abbas, Mohammed Gollapalli, Rashad Ahmed, Shabib Aftab, Munir Ahmad, Muhammad Adnan Khan, Amir Mosavi

**Affiliations:** 1 Department of Computer Science, College of Computer Science and Information Technology, Imam Abdulrahman Bin Faisal University, P.O. Box 1982, Dammam 31441, Saudi Arabia; aaurrahman@iau.edu.sa; 2 School of Computer Science, National College of Business Administration and Economics, Lahore 54000, Pakistan; dr.sagheer@ncbae.edu.pk (S.A.); shabib.aftab@ncbae.edu.pk (S.A.); munir@ncbae.edu.pk (M.A.); 3 Department of Computer Information Systems, College of Computer Science and Information Technology, Imam Abdulrahman Bin Faisal University, P.O. Box 1982, Dammam 31441, Saudi Arabia; magollapalli@iau.edu.sa; 4 ICS Department, King Fahd University of Petroleum and Minerals, Dhahran 31261, Saudi Arabia; othmanr@kfupm.edu.sa; 5 Department of Computer Science, Virtual University of Pakistan, Lahore 54000, Pakistan; 6 Department of Software, Gachon University, Seongnam 13120, Korea; 7 John von Neumann Faculty of Informatics, Obuda University, 1034 Budapest, Hungary; mosavi.amirhosein@uni-nke.hu; 8 Institute of Information Engineering, Automation and Mathematics, Slovak University of Technology in Bratislava, 81107 Bratislava, Slovakia; 9 Faculty of Civil Engineering, TU-Dresden, 01062 Dresden, Germany

**Keywords:** rainfall, rainfall prediction, machine learning, data fusion, fuzzy system, smart cities, big data, hydrological model, information systems, precipitation

## Abstract

Precipitation in any form—such as rain, snow, and hail—can affect day-to-day outdoor activities. Rainfall prediction is one of the challenging tasks in weather forecasting process. Accurate rainfall prediction is now more difficult than before due to the extreme climate variations. Machine learning techniques can predict rainfall by extracting hidden patterns from historical weather data. Selection of an appropriate classification technique for prediction is a difficult job. This research proposes a novel real-time rainfall prediction system for smart cities using a machine learning fusion technique. The proposed framework uses four widely used supervised machine learning techniques, i.e., decision tree, Naïve Bayes, K-nearest neighbors, and support vector machines. For effective prediction of rainfall, the technique of fuzzy logic is incorporated in the framework to integrate the predictive accuracies of the machine learning techniques, also known as fusion. For prediction, 12 years of historical weather data (2005 to 2017) for the city of Lahore is considered. Pre-processing tasks such as cleaning and normalization were performed on the dataset before the classification process. The results reflect that the proposed machine learning fusion-based framework outperforms other models.

## 1. Introduction

Knowledge extraction from time series data has become a widely explored research area [1,2]. Data which are collected with time stamps in a specific pattern are called time series data [3,4,5]. This type of time-oriented data is collected with a specific time interval, such as on an hourly, daily, or weekly basis. Time series data can be utilized effectively to make predictions in various areas and domains, including foreign currency rates, stock market trends, energy consumption estimations, and climate change. Machine learning and data mining techniques can be utilized to extract the hidden patterns from historical data in order to forecast the future trend [1,2,5,6]. Weather forecasting on the basis of historical data is a complex but very beneficial task [7] which comes with several problems that need to be solved in order to achieve optimal results. Weather-related data consists of various attributes or features such as temperature, pressure, humidity, and wind speed. Machine learning techniques tend to predict future weather conditions by using hidden patterns and relations among the features of historical weather data [2]. Precipitation prediction is one of the crucial stages of the weather forecasting process. A smart city is a place where all the community elements, including people and devices, are connected with advanced technologies. In these urban areas, data are collected from citizens as well as from buildings through sensors and electronic devices; the data is then used to manage resources, services, and assets effectively and efficiently. In such technologically advanced cities, data are processed, analyzed, and then used to monitor and manage various systems and activities; as such, data are considered to be very important. The data collected from different sources in smart cities are ultimately used in various automatic systems, including traffic and transportation systems, water supply networks, power plants, waste collection and disposal systems, crime detection systems, education systems, and other community services. The use of machine learning and artificial intelligence techniques is considered to be a crucial element in the services and products of smart cities. Weather forecasting is necessary for the citizens of smart cities so that people can plan their activities according to the predicted weather. In particular, accurate and timely rainfall prediction in smart cities can be quite helpful for arranging planning and security measures in advance for flight operations, agricultural tasks, water reservoir systems, and constructions and transportation activities [2,8,9]. A red alert in advance in the case of extreme rainfall can save the citizens of smart cities from potentially life-threatening situations.

This research presents a rainfall prediction framework using a machine learning fusion technique for smart cities. The real-time weather data are collected from multiple sensors located in various vital locations of the city. Four classification techniques are used in the proposed framework for fusion, including Decision Tree (DT), Naïve Bayes (NB), K-Nearest Neighbors (KNN), and Support Vector Machines (SVM) [10,11,12]. To achieve high accuracy, a fuzzy logic-based layer is included in the proposed framework, which integrates the predictive performance of used classification techniques. These algorithms belong to a supervised class of data mining, in which training is required first with pre-classified data, where classification rules are built and then applied to the input dataset (test data) [13,14]. A weather forecasting website [15] is used to extract the relevant data. The extracted data spans 12 years, from December 2005 to November 2017, and consists of various attributes, including maximum temperature, minimum temperature, and relative humidity. The dataset used in this research has already been used by us in [1]. In this research, a framework consisting of multiple stages has been developed for effective predictions. The framework begins with a pre-processing phase which deals with the cleaning and normalization of data [16,17]. The cleaning process deals with the outliers and missing values, whilst the normalization process keeps the feature values within a particular range. The cleaned and normalized values then go to the classification stage where DT, NB, KNN, and SVM are tuned and then used for prediction. The predicted results from these machine learning techniques are given to fusion layer as input, where fuzzy logic-based rules are used for final prediction. The fused model is then stored in the cloud for prediction using real-time weather data.

## 2. Literature Review

Improving the accuracy of machine learning techniques on weather forecasting has been the primary concern of many researchers over the last two decades. Some of the related studies are discussed here. In [18], researchers presented an ANN-based technique to predict atmospheric conditions. The dataset used for prediction consisted of various weather attributes, e.g., humidity, temperature, and wind speed. The proposed technique integrated the Back Propagation Network and Hopfield Network in such a way that the output of BPN is given to the HN as input. This technique works by exploring the non-linear relationship between historical weather attributes. In [19], researchers used ANN to predict the monthly average rainfall of monsoon weather in India. A dataset covering a period of 8 months each year was used for prediction. The selected months were considered to have a high probability rainfall. Three types of different networks were used for performance analysis: Feed Forward Back Propagation, Layer Recurrent, and Cascaded Feed Forward Back Propagation. According to the results, Feed Forward Back Propagation outperformed the others. In [20], researchers proposed a rainfall prediction technique which used genetic algorithms for feature selection and Naïve Bayes as a predictive algorithm. The proposed solution had two steps: the first step deals with the prediction of rainfall (whether it will rain or not), and the second step classifies the rainfall as light, moderate, or strong. In [21], researchers presented a framework consisting of deep neural networks to predict weather changes over the next 24 h. For prediction, they used a dataset covering 30 years, from 1983 to 2012, obtained from Hong Kong Observatory (HKO). The dataset consisted of four weather attributes: temperature, dew point, mean sea level pressure, and wind speed. According to the results, DNNs provided a good feature space for weather datasets. In [22], researchers presented a new pre-processing technique by using moving average and singular spectrum analysis. The proposed approach can be applied on the classes of training data in order to transform it into low, medium, and high categories. Prediction was performed using an Artificial Neural Network (ANN). Two daily rainfall datasets—Zhenshui and Da’ninghe water sheds in China—were used for experiment.

In [23], researchers proposed a hybrid method for rainfall forecasting by integrating feature extraction and prediction techniques. The dataset used for the experiment was obtained from the National Oceanic and Atmospheric Administration (NOAA); it spanned more than 50 years and consisted of various weather features such as humidity, pressure, temperature, and wind speed. A Neural Network was used to classify the instances into low, medium, and high classes based on a pre-defined training set. In [24], researchers presented a data-intensive model for rainfall prediction using a Bayesian modeling approach. For the experiment, the dataset was collected from the Indian Meteorological Department, and from 36 attributes, the 7 most relevant attributes were selected. Before the prediction, pre-processing and transformation steps were performed for smooth processing. The proposed approach showed good accuracy for rainfall prediction, using moderate computing resources compared to meteorological centers using high-performance computing power for weather predictions. In [25], researchers compared different machine learning techniques for the prediction of rainfall in Malaysia. The mining techniques included Naïve Bayes, Neural Network, SVM, Decision Tree, and Random Forest. Pre-processing was performed on the dataset to fill the missing values and to remove the noise before classification. Random Forest outperformed the others; it correctly classified a large number of instances with a small portion of training data. In [26], the technique of Clusterwise Linear Regression was employed, which involved integrating the clustering and regression methods. The proposed CLR technique predicted the monthly rainfall in Victoria, Australia. The used dataset was obtained from eight geographically diverse weather stations, spanning from 1889 to 2014. The performance was compared with other published techniques; it was shown that in most of the locations, CLR performed better than others. In [27], researchers compared “Markov Chain extended with rainfall prediction” with other widely used data mining techniques, including Radial Basis, Neural Networks, Genetic Programming, Support Vector Regression, M5 Rules, k-Nearest Neighbors, and M5 Model trees. A dataset obtained from 42 cities was used for the experiment. The results showed that the Markov Chain technique can be outperformed by machine learning techniques. The correlation between weather-related attributes and accuracy has also been noted.

In [28], two forecasting models were developed for rainfall prediction: the first predicted for 1 month ahead, whilst the second predicted for 2 months ahead by using ANN. A dataset from several locations of north India was used for the experiment. The model integrated the Feed Forward Neural Network with Back Propagation technique, along with the Levenberg–Marquardt training function. The performance was analyzed in terms of Mean Square Error and Magnitude of Relative Error. According to the results, the 1-month ahead forecasting model outperformed the 2-month model. In [29], researchers proposed a framework named the Wavelet Neural Network (WNN) to predict the rainfall. The proposed solution integrated ANN with the wavelet technique. Both models (ANN and WNN) were used for prediction by using rainfall historical data from the Darjeeling rain gauge station, situated in West Bengal, India. According to the results, WNN outperformed ANN. In [30], researchers presented an SVM-based application for the prediction of weather. A time series dataset related to the past n days from a location was analyzed, and then the maximum temperature of that location for the next day was predicted. By using optimal values of the kernel function, the performance of the proposed application was evaluated and found to outperform Multi-Layer Perceptron (MLP), trained with a back-propagation algorithm. To train the SVM, a nonlinear regression method was found to be suitable. In [31], researchers presented an advanced statistical technique for solar power forecasting based on an artificial intelligence approach. The proposed technique requires several features as input, such as past power measurements and meteorologically related forecasts. The required metrological data included solar irradiance, relative humidity, and temperature. A SOM (Self organized map) was trained to classify the local weather 24 h in advance with the help of online meteorological services. The proposed method was considered to be suitable for the forecasting of 24 h ahead power output of a PV (photovoltaic) system, as well as for trading in electricity markets of PV power system operators.

In [32], researchers presented the technique of modular-based Support Vector Machine (SVM) to predict and simulate rainfall prediction. The proposed technique consisted of several steps, such as the generation of training sets with the bagging sampling technique, training of SVM kernel function, selection of SVM combination members with the PLS (Partial Least Square) technique, and production of *ν*–SVM. The proposed technique was used for monthly rainfall prediction in Guangxi, China and outperformed other models.

Table 1 summarizes the previously published related work. Previously, most researchers used supervised machine learning classifiers in order to predict rainfall by exploring hidden patterns in historical data. The researchers mostly used more than one technique in the proposed frameworks: one for feature selection and one for classification and prediction. Rainfall forecasting using time series weather data has also been widely explored by researchers. This research proposes a framework for rainfall prediction, particularly for smart cities, where real-time weather data is continuously collected from specific weather sensors. Moreover, to increase the performance, the predictive accuracy of four classifiers (DT, NB, KNN, and SVM) is integrated with the help of fuzzy logic.

## 3. Materials and Methods

**Figure 1:**
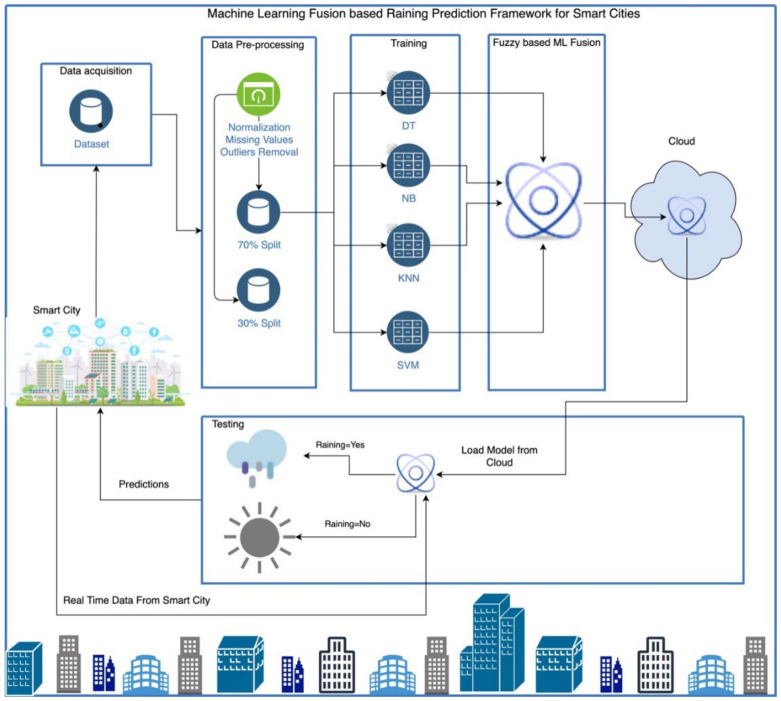
Proposed framework.

This research purposes a rainfall prediction framework (Figure 1) using a machine learning fusion technique for smart cities. The proposed framework mainly consists of two layers: training and testing. Both of these layers further include multiple stages. The first stage of the training layer deals with the extraction of weather attributes from technologically advanced sensors in the smart city. However, in this research, we have extracted a real-time pre-labeled dataset of rainfall prediction from a weather forecasting website [15] of the city of Lahore. The dataset consists of 25,919 instances and 11 features, out of which 10 features are independent and 1 is dependent (output class). The data pre-processing stage consists of three activities: (1) cleaning, (2) normalization, and (3) splitting. The data cleaning process aims to remove the missing values in the dataset by using the technique of mean imputation. The normalization technique brings the attribute values within a particular range. These cleaning and normalization activities aid the classifiers in obtaining maximum accuracy. In the third activity of the pre-processing stage, cleaned and normalized data is divided into two subsets: training data and test data, with a 70:30 ratio of class split rule. After performing the tasks of pre-processing activities, the dataset is ready for the stage of classification, where training and test datasets are both given as input to four classification techniques (DT, NB, KNN, and SVM). All of these algorithms are optimized iteratively during training and testing in order to achieve higher accuracy. After the classification process, the trained models are given as input to the fuzzy layer, which deals with the development and implementation of fuzzy logic for final prediction.

The fused proposed prediction model after training is stored in cloud storage so that it can be used for later prediction by using real-time testing data. Conditions (if–then rules) used in the fuzzy logic of the proposed framework are given below:

- IF (DT is yes and NB is yes and KNN is yes and SVM is yes) THEN (Rainfall is yes)
- IF (DT is yes and NB is yes and KNN is yes and SVM is no) THEN (Rainfall is yes)
- IF (DT is yes and NB is yes and KNN is no and SVM is yes) THEN (Rainfall is yes)
- IF (DT is yes and NB is yes and KNN is no and SVM is no) THEN (Rainfall is yes)
- IF (DT is yes and NB is no and KNN is yes and SVM is yes) THEN (Rainfall is yes)
- IF (DT is yes and NB is no and KNN is yes and SVM is no) THEN (Rainfall is yes)
- IF (DT is yes and NB is no and KNN is no and SVM is yes) THEN (Rainfall is yes)
- IF (DT is yes and NB is no and KNN is no and SVM is no) THEN (Rainfall is no)
- IF (DT is no and NB is yes and KNN is yes and SVM is yes) THEN (Rainfall is yes)
- IF (DT is no and NB is yes and KNN is yes and SVM is no) THEN (Rainfall is no)
- IF (DT is no and NB is yes and KNN is no and SVM is yes) THEN (Rainfall is no)
- IF (DT is no and NB is yes and KNN is no and SVM is no) THEN (Rainfall is no)
- IF (DT is no and NB is no and KNN is yes and SVM is yes) THEN (Rainfall is no)
- IF (DT is no and NB is no and KNN is yes and SVM is no) THEN (Rainfall is no)
- IF (DT is no and NB is no and KNN is no and SVM is yes) THEN (Rainfall is no)
- IF (DT is no and NB is no and KNN is no and SVM is no) THEN (Rainfall is no)

**Figure 2:**
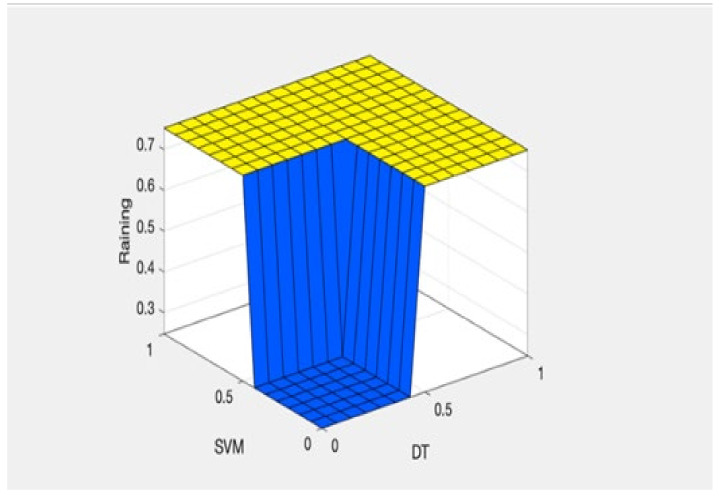
Rule surface of proposed fused technique for SVM and DT.

**Figure 3:**
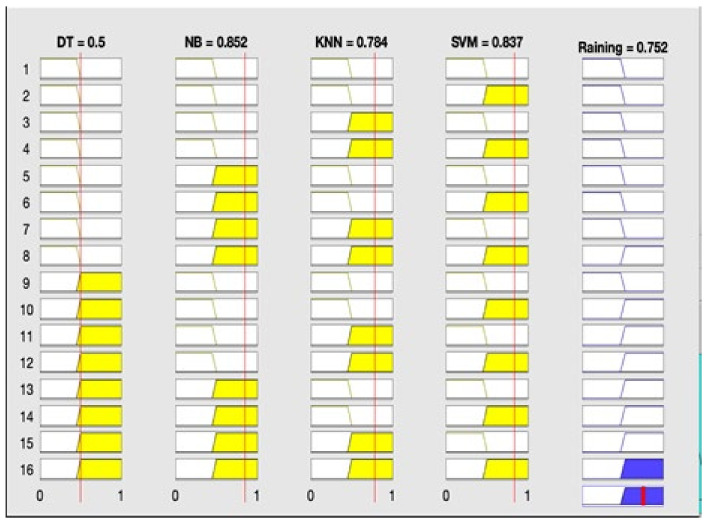
Result of proposed framework: rainfall = yes.

**Figure 4:**
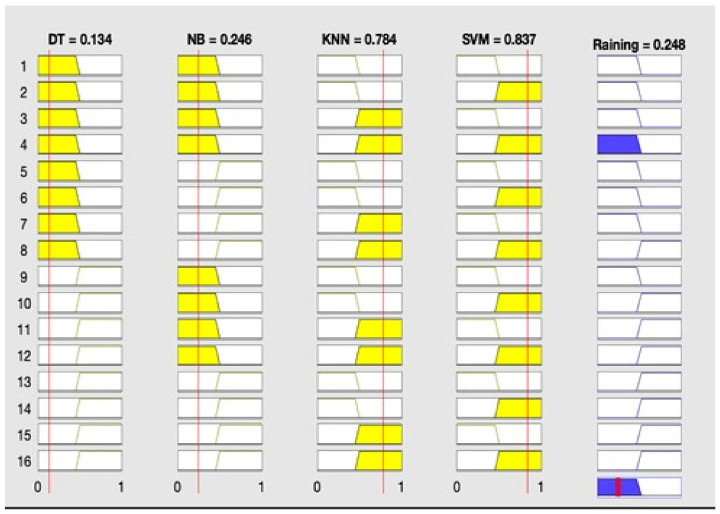
Result of proposed framework: rainfall = no.

It can be observed from the developed fuzzy rules that if any of three classification techniques predict one result (either rain or no rain), the same result will be predicted by the proposed fused technique. Figure 2 reflects the proposed fused technique rule surface of rainfall prediction on the basis of SVM and DT. If both of these classification techniques predict ‘rainfall = yes’, then the result of the fused machine learning technique will also be ‘rainfall = yes’, and if both of these techniques predict ‘rainfall = no’, then the proposed technique will also predict ‘rainfall = no’. It is shown in Figure 3 that if NB, KNN, and SVM predict ‘rainfall = yes’, then the proposed fused technique will also predict ‘rainfall = yes’. Figure 4 shows that if DT and NB predict ‘rainfall = no’, even if KNN and SVM predict ‘rainfall = yes’, then the result of the proposed technique will still be ‘rainfall = no’. The membership functions of the proposed fuzzy rules are shown in Table 2. The testing layer of the proposed framework is responsible for predicting rainfall by using real-time weather data. The fuzzy trained model from the cloud is used for this purpose, which takes the input of real-time weather data as test data.

## 4. Results and Discussion

The proposed framework is implemented on a real-time rainfall dataset of the city of Lahore, extracted from a weather forecasting website [15]. The dataset used in this research spans over 12 years (2005 to 2017) and consists of 25,919 instances and 11 features (Table 3). First, 10 features are the independent features, which are given as input to the proposed framework in order to predict the 11th feature, which is the output class (dependent feature). The output class indicates whether there will be rainfall or not. If the predicted feature has a value of 1, then will be a rainy day; if the value is 0, then it will be no rainfall. The dataset is divided into two parts: 70% of the data is reserved for training (18,143), and 30% of the data is reserved for testing (7776). The activities of the pre-processing stage, including cleaning and normalization, are performed on the rainfall dataset before the classification stage. To predict, four classification techniques are used: DT, NB, KNN, and SVM. These classification techniques are optimized iteratively until maximum accuracy is achieved.

The statistical measures used to analyze the predictive performance of the proposed fused framework as well as of other classification techniques are discussed below.

In the formulas given below, OR_0_ represents predicted negatives, OR_1_ represents predicted positives, ER_0_ represents expected negatives, and ER_1_ represents expected positives.

Miss rate is the probability of true positives and true negatives being missed in the experiment [1,10,34].

(1) $$\mathrm{Miss}\;\mathrm{rate}=\frac{\left({\mathrm{OR}}_{1}/{\mathrm{ER}}_{0}+{\;\mathrm{OR}}_{0}/{\mathrm{ER}}_{1}\right)}{{\mathrm{ER}}_{0}+{\;\mathrm{ER}}_{1}}$$

Accuracy reflects the number of correctly classified instances out of total instances [10,13,34].

(2) $$\mathrm{Accuracy}=\frac{\left({\mathrm{OR}}_{0}/{\mathrm{ER}}_{0}+{\;\mathrm{OR}}_{1}/{\mathrm{ER}}_{1}\right)}{{\mathrm{ER}}_{0}+{\;\mathrm{ER}}_{1}}$$

The positive and negative predictive values are the proportions of positive and negative results to the true positive and true negative results, respectively [1,34].

(3) $$\mathrm{Positive}\;\mathrm{Prediction}\;\mathrm{Value}=\frac{{\mathrm{OR}}_{1}/{\mathrm{ER}}_{1}}{\left({\mathrm{OR}}_{1}/{\mathrm{ER}}_{1}+{\;\mathrm{OR}}_{0}/{\mathrm{ER}}_{1}\right)}$$

(4) $$\mathrm{Negative}\;\mathrm{Prediction}\;\mathrm{Value}=\frac{{\mathrm{OR}}_{0}/{\mathrm{ER}}_{0}}{\left({\mathrm{OR}}_{0}/{\mathrm{ER}}_{0}+{\;\mathrm{OR}}_{1}/{\mathrm{ER}}_{0}\right)}$$

(5) $$\mathrm{Specificity}=\frac{{\mathrm{OR}}_{0}/{\mathrm{ER}}_{0}}{\left({\mathrm{OR}}_{0}/{\mathrm{ER}}_{0}+{\;\mathrm{OR}}_{0}/{\mathrm{ER}}_{1}\right)}$$

Sensitivity reflects how well the proposed model can detect positive instances [10,34].

(6) $$\mathrm{Sensitivity}=\frac{{\mathrm{OR}}_{1}/{\mathrm{ER}}_{1}}{\left({\mathrm{OR}}_{1}/{\mathrm{ER}}_{0}+{\;\mathrm{OR}}_{1}/{\mathrm{ER}}_{1}\right)}$$

The false positive rate reflects the ratio between false positives and the total number of instances which are actually negative [34].

(7) False Positive Ratio=1−Specificity

(8) False Negative Ratio=1−Sensitivity

(9) $$\mathrm{Likelihood}\;\mathrm{Ratio}\;\mathrm{Positive}=\frac{\mathrm{Sensitivity}}{\left(1-\mathrm{Specificity}\right)}$$

(10) $$\mathrm{Likelihood}\;\mathrm{Ratio}\;\mathrm{Negative}=\frac{\left(1-\;\mathrm{Sensitivity}\right)}{\mathrm{Specificity}}$$

First, the DT is used for the prediction of rainfall. Then, 70% of the dataset (consisting of 18,143 instances) is used for training; the remaining 30% of the dataset (consisting of 7776 instances) is used for testing. From the 18,143 instances reserved for training, 16,577 were negative and 1566 were positive. During the training with DT, 16,456 instances from 16577 were classified as negative, and 372 instances were classified as positive from 1566 instances. After analyzing the achieved results compared with expected results during the training process (Table 4), it is calculated that we achieved an accuracy of 92.8% and a miss rate of 7.2%. On the other hand, during the testing process of DT, 7036 records were classified as negative from 7105, and 155 records were classified as positive from 671 records (as shown in Table 5). The accuracy achieved in DT testing was 92.48%, with a miss rate of 7.52%.

During the training with NB, 16,176 instances were classified as negative from 16,577 instances, and 280 instances were classified as positive from 1566 instances (as shown in Table 6). We achieved an accuracy of 90.7% and a miss rate of 9.3% for training with NB. During testing with NB, 6937 instances were classified as negative from 7105 instances, and 116 instances were classified as positive from 671 instances (as shown in Table 7). The accuracy achieved for testing with NB was 90.7%, with a miss rate of 9.3%, when we compared the expected output with the output results.

During the training process with KNN, 16,481 instances were classified as negative from 16577 instances, and 316 instances were classified as positive from 1566 instances. From the comparison of expected output with the achieved output in training with KNN (Table 8), it can be observed that we achieved an accuracy of 92.6% and a miss rate of 7.4%. During the testing with KNN, 7050 instances were classified as negative from 7105 instances, and 143 instances were classified as positive from 671 instances (as shown in Table 9). After analyzing the expected output with the achieved output, we determined that we obtained an accuracy of 92.5% and a miss rate of 7.5% for the testing process with KNN.

During the process of training with SVM, 16544 instances were classified as negative from 16,577 instances, and 182 instances were classified as positive from 1566 instances (as shown in Table 10). While performing a comparative analysis of expected output result with the achieved output result, we determined that we obtained an accuracy of 92.2% in training, with a miss rate of 7.8%. During testing, 7086 instances were classified as negative from 7105 instances, and 75 instances were classified as positive from 671 instances (as shown in Table 11). In the testing process with SVM, we achieved an accuracy of 92.1% and a miss rate of 7.9%.

Finally, all of the instances from the testing data are given to the fuzzy system as input for the final prediction. The input to the fuzzy system includes test data along with the output class, and the predictions of used classifiers. The proposed fused machine learning-based fuzzy system classified 7063 instances as negative from 7105 instances, and 228 instances as positive from 671 instances (as shown in Table 12). While comparing the output result of the fuzzy system with the expected result, we determined that we achieved an accuracy of 94% and a miss rate of 6%. Table 13 displays detailed results for training and test data of all of the used classification techniques (DT, NB, KNN, SVM) and the proposed fused machine learning technique. It can be observed that the proposed fused technique performed well compared to all four of the used machine learning techniques. Table 14 shows a comparative analysis of the proposed fused machine learning technique with the previously published techniques for rainfall prediction in terms of accuracy and miss rate. The proposed fused model is compared with KNN [6], Naïve Bayes [6], CART [6], PRNN [6], Bayesian [24], INBC [5], and DT-SLIQ [33]. It can be seen that the proposed fused model performed better than the other techniques. The proposed machine learning fusion based framework can be incorporated into smart cities for accurate rainfall prediction. The proposed framework will be linked to highly sensitive and technologically advanced weather sensors. These sensors will provide weather data to the system on a continuous basis, which will be used for real-time rainfall prediction.

## 5. Conclusions

Rainfall prediction with maximum accuracy is a challenging task of the weather forecasting process. The use of machine learning techniques has increased the accuracy of rainfall prediction systems by exploring the hidden patterns of historical weather data. A novel and real-time rainfall prediction system is proposed by this research for smart cities by using machine learning fusion. The proposed framework would extract the real-time feature-based weather data from highly sensitive and technologically advanced weather sensors for real-time rainfall prediction. The prediction accuracy of four supervised machine learning techniques are integrated in the proposed framework. The used machine learning techniques are Decision Tree, Naïve Bayes, K-Nearest Neighbors, and Support Vector Machines. The prediction accuracy of the used machine learning techniques are fused using fuzzy logic. For the experiment, 12 years of historical weather data (from 2005 to 2017) for the city of Lahore was extracted from a weather forecasting website, consisting of various weather-related features. To improve the accuracy of classification and prediction, pre-processing activities were performed on the extracted dataset, including cleaning and normalization. The results clearly show the effectiveness of the proposed framework by reflecting the higher accuracy compared to other modern techniques. The proposed machine learning fusion-based rainfall prediction system has one limitation besides the many advantages. If due to any reason, the data which will be used for prediction is compromised, then the prediction cannot be trusted. Any type of malfunction in the weather sensor can also compromise the accuracy of the proposed rainfall prediction system. Therefore, a monitoring system to check the working of weather sensors has also be incorporated along with the information security system, which will ensure the integrity of the data until it is used for prediction. The framework presented in this research will be extended in the future by exploring the fusion of ensemble machine learning techniques on more diverse datasets. Moreover, an appropriate feature selection technique would also be an effective addition to the system, which will ensure cost-effective prediction. Besides rainfall prediction, machine learning fusion will also be used for temperature prediction in order to efficiently utilize clean solar energy. Efforts will be made to incorporate the various flavors of Artificial Neural Networks in the weather forecasting process, such as Multi-Layer Perceptron (MLP) and Long Short-Term Memory (LSTM) networks.

## Figures and Tables

**Table 1:** Summary of previous related work.

Reference	Method	Dataset	Dataset Duration	Accuracy %
D. Gupta et al. [6]	ANN-based classification model, with 10 hidden layers	Public	18 years	82.1
D. Gupta et al. [6]	Classification and Regression Tree-based Prediction	Public	18 years	80.3
D. Gupta et al. [6]	K nearest neighbor-based prediction, with k = 22	Public	18 years	80.7
J. Joseph et al. [23]	ANN-based hybrid technique, integrating classification and clustering techniques	Private	4 months	87
V.B. Nikam et al. [24]	Feature selection-based Bayesian classification model	Public	6 months	91
N. Prasad et al. [33]	Decision Tree-based supervised learning in quest (SLIQ)	Public	14 years	72.3

**Table 2:** Graphical representation of MF.

Input/Output	Membership Functions	Graphical Representation of MF
$\mathrm{DT}=\mu_{DT}\left(dt\right)$	$\mu_{DT_{y}}\left(dt\right)=\left\{\max\left(\min\left(1,\frac{0.5-dt}{0.05}\right),0\right)\right\}$ $\mu_{DT_{n}}\left(dt\right)=\left\{\max\left(\min\left(\frac{dt-0.45}{0.05},1\right),0\right)\right\}$	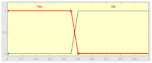
$\mathrm{NB}=\mu_{NB}\left(nb\right)$	$\mu_{NB_{y}}\left(nb\right)=\left\{\max\left(\min\left(1,\frac{0.5-nb}{0.05}\right),0\right)\right\}$ $\mu_{NB_{n}}\left(nb\right)=\left\{\max\left(\min\left(\frac{nb-0.45}{0.05},1\right),0\right)\right\}$	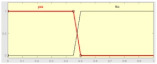
$\mathrm{KNN}=\left(knn\right)$	$\mu_{KNN_{y}}\left(knn\right)=\left\{\max\left(\min\left(1,\frac{0.5-knn}{0.05}\right),0\right)\right\}$ $\mu_{KNN_{n}}\left(knn\right)=\left\{\max\left(\min\left(\frac{knn-0.45}{0.05},1\right),0\right)\right\}$	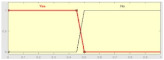
$\mathrm{SVM}=\mu_{SVM}\left(svm\right)$	$\mu_{SVM_{y}}\left(svm\right)=\left\{\max\left(\min\left(1,\frac{0.5-svm}{0.05}\right),0\right)\right\}$ $\mu_{SVM_{n}}\left(svm\right)=\left\{\max\left(\min\left(\frac{svm-0.45}{0.05},1\right),0\right)\right\}$	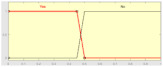
$\mathrm{Raining}=\mu_{R}\left(r\right)$	$\mu_{R_{y}}\left(r\right)=\left\{\max\left(\min\left(1,\frac{0.5-r}{0.05}\right),0\right)\right\}$ $\mu_{R_{n}}\left(r\right)=\left\{\max\left(\min\left(\frac{r-0.45}{0.05},1\right),0\right)\right\}$	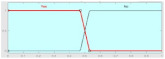

**Table 3:** Dataset attributes.

Attribute Name	Attribute Type	Measurement
Temperature	Continuous	Degrees Celsius
Visibility	Continuous	Kilometers
Dew Point Temperature	Continuous	Degrees Celsius
Atmospheric Pressure (sea level)	Continuous	Millimeters of Mercury
Atmospheric Pressure (weather station)	Continuous	Millimeters of Mercury
Relative Humidity	Continuous	Percentage
Pressure Tendency	Continuous	Millimeters of Mercury
Maximum Temperature	Continuous	Degrees Celsius
Minimum Temperature	Continuous	Degrees Celsius
Mean Wind Speed	Continuous	Meters per Second

**Table 4:** DT Training Results.

N = 18,143 (No of Samples)		Output Result (OR_0_, OR_1_)	
INPUT	Expected Result (ER_0,_ ER_1_)	OR_0_ (Negative-0)	OR_1_ (Positive-1)
	ER_0_ = 16,577 (Negative-0)	16456	121
	ER_1_ = 1566 (Positive-1)	1194	372

**Table 5:** DT Testing results.

N = 7776 (No of Samples)		Output Result (OR_0_, OR_1_)	
INPUT	Expected Result (ER_0,_ ER_1_)	OR_0_ (Negative-0)	OR_1_ (Positive-1)
	ER_0_ = 7105 (Negative-0)	7036	69
	ER_1_ = 671 (Positive-1)	516	155

**Table 6:** Naïve Bayes training results.

N = 18,143 (No of Samples)		Output Result (OR_0_, OR_1_)	
INPUT	Expected Result (ER_0,_ ER_1_)	OR_0_ (Negative-0)	OR_1_ (Positive-1)
	ER_0_ = 16,577 (Negative-0)	16176	401
	ER_1_ = 1566 (Positive-1)	1286	280

**Table 7:** Naïve Bayes testing results.

N = 7776 (No of Samples)		Output Result (OR_0_, OR_1_)	
INPUT	Expected Result (ER_0_, ER_1_)	OR_0_ (Negative-0)	OR_1_ (Positive-1)
	ER_0_ = 7105 (Negative-0)	6937	168
	ER_1_ = 671 (Positive-1)	555	116

**Table 8:** KNN training results.

N = 18,143 (No of Samples)		Output Result (OR_0_, OR_1_)	
INPUT	Expected Result (ER_0_, ER_1_)	OR_0_ (Negative-0)	OR_1_ (Positive-1)
	ER_0_ = 16,577 (Negative-0)	16481	96
	ER_1_ = 1566 (Positive-1)	1250	316

**Table 9:** KNN testing results.

N = 7776 (No of Samples)		Output Result (OR_0_, OR_1_)	
INPUT	Expected Result (ER_0_, ER_1_)	OR_0_ (Negative-0)	OR_1_ (Positive-1)
	ER_0_ = 7105 (Negative-0)	7050	55
	ER_1_ = 671 (Positive-1)	528	143

**Table 10:** SVM training results.

N = 18,143 (No of Samples)		Output Result (OR_0_, OR_1_)	
INPUT	Expected Result (ER_0_, ER_1_)	OR_0_ (Negative-0)	OR_1_ (Positive-1)
	ER_0_ = 16577 (Negative-0)	16544	33
	ER_1_ = 1566 (Positive-1)	1384	182

**Table 11:** SVM testing results.

N = 7776 (No of Samples)		Output Result (OR_0_, OR_1_)	
INPUT	Expected Result (ER_0_, ER_1_)	OR_0_ (Negative-0)	OR_1_ (Positive-1)
	ER_0_ = 7105 (Negative-0)	7086	19
	ER_1_ = 671 (Positive-1)	596	75

**Table 12:** Fused ML testing results.

N = 7776 (No of Samples)		Output Result (OR_0_, OR_1_)	
INPUT	Expected Result (ER_0_, ER_1_)	OR_0_ (Negative-0)	OR_1_ (Positive-1)
	ER_0_ = 7105 (Negative-0)	7063	42
	ER_1_ = 671 (Positive-1)	443	228

**Table 13:** Results of machine learning algorithms.

ML Algorithm	Task	Specificity	Sensitivity	False Positive Value	False Negative Value	Likelihood Ratio Positive	Likelihood Ratio Negative	Positive Prediction Value	Negative Prediction Value	Accuracy	Miss Rate
Decision Tree	Training	0.99	0.24	0.00	0.76	32.54	0.77	0.75	0.93	0.91	0.07
	Testing	0.99	0.23	0.01	0.77	23.79	0.78	0.69	0.93	0.92	0.07
Naïve Bayes	Training	0.98	0.18	0.02	0.82	7.39	0.84	0.41	0.93	0.90	0.09
	Testing	0.98	0.17	0.02	0.83	7.31	0.85	0.41	0.93	0.90	0.09
KNN	Training	0.99	0.20	0.00	0.80	34.84	0.80	0.77	0.91	0.93	0.07
	Testing	0.99	0.21	0.00	0.79	27.53	0.79	0.72	0.93	0.93	0.07
SVM	Training	0.99	0.12	0.00	0.88	58.38	0.89	0.85	0.92	0.92	0.08
	Testing	0.99	0.11	0.00	0.89	41.80	0.89	0.80	0.92	0.92	0.08
Proposed Fussed ML	Testing	0.99	0.34	0.01	0.66	57.48	0.66	0.84	0.94	0.94	0.06

**Table 14:** Comparison of proposed fusion model with previously published approaches.

Algorithm	Accuracy Rate	Miss Rate
KNN (K = 22) [6]	80.7	19.3
Naïve Bayes [6]	78.9	21.1
CART (pruning) [6]	80.3	19.7
PRNN (10 neuron) [6]	82.1	17.9
Bayesian [24]	91	9
INBC [5]	90	10
DT-SLIQ [33]	72.3	27.7
Proposed Fused ML	94	6

## Data Availability

The simulation files/data used to support the findings of this study are available from the corresponding author upon request.
